# Sequence-Defined Nanotubes Assembled from IR780-Conjugated Peptoids for Chemophototherapy of Malignant Glioma

**DOI:** 10.34133/2021/9861384

**Published:** 2021-05-16

**Authors:** Xiaoli Cai, Mingming Wang, Peng Mu, Tengyue Jian, Dong Liu, Shichao Ding, Yanan Luo, Dan Du, Yang Song, Chun-Long Chen, Yuehe Lin

**Affiliations:** ^1^School of Mechanical and Materials Engineering, Washington State University, PO Box 642920 Pullman, Washington 99164, USA; ^2^Physical Sciences Division, Pacific Northwest National Laboratory, Richland, WA 99352, USA; ^3^Department of Mechanical Engineering and Materials Science and Engineering Program, State University of New York, Binghamton, New York 13902, USA

## Abstract

Near-infrared (NIR) laser-induced phototherapy through NIR agents has demonstrated the great potential for cancer therapy. However, insufficient tumor killing due to the nonuniform heat or cytotoxic singlet oxygen (^1^O_2_) distribution over tumors from phototherapy results in tumor recurrence and inferior outcomes. To achieve high tumor killing efficacy, one of the solutions is to employ the combinational treatment of phototherapy with other modalities, especially with chemotherapeutic agents. In this paper, a simple and effective multimodal therapeutic system was designed *via* combining chemotherapy, photothermal therapy (PTT), and photodynamic therapy (PDT) to achieve the polytherapy of malignant glioma which is one of the most aggressive tumors in the brain. IR-780 (IR780) dye-labeled tube-forming peptoids (PepIR) were synthesized and self-assembled into crystalline nanotubes (PepIR nanotubes). These PepIR nanotubes showed an excellent efficacy for PDT/PTT because the IR780 photosensitizers were effectively packed and separated from each other within crystalline nanotubes by tuning IR780 density; thus, a self-quenching of these IR780 molecules was significantly reduced. Moreover, the efficient DOX loading achieved due to the nanotube large surface area contributed to an efficient and synergistic chemotherapy against glioma cells. Given the unique properties of peptoids and peptoid nanotubes, we believe that the developed multimodal DOX-loaded PepIR nanotubes in this work offer great promises for future glioma therapy in clinic.

## 1. Introduction

Photobased therapies such as photothermal therapy (PTT) and photodynamic therapy (PDT) have recently been developed as promising therapeutic strategies for antitumor therapy because of their minimal invasiveness, low side effects, and high specificity [1–6]. For PTT and PDT, they usually utilize the near-infrared (NIR) laser-induced capability of photothermal agents and photosensitizers (PSs) to generate heat or cytotoxic singlet oxygen (^1^O_2_), which subsequently causes cell apoptosis [7–10]. However, because of the nonuniform distribution of the near-infrared agents at the tumor site, the heat or cytotoxic ^1^O_2_ distribution over tumors is also nonuniform. It leads to the insufficient tumor cell killing, incomplete ablation, tumor recurrence, and inferior outcomes [11, 12]. Recently, the combination of phototherapy and chemotherapy (chemophototherapy, CPT) has been designed and proved for a synergistic cancer treatment to enhance the cancer treatment efficacy and reduce the toxicities and drug resistance during the chemotherapy [13–15]. For example, a tumor microenvironment- (TME-) responsive system PCPT_SS_/IR820 was designed by Shi et al.'s group for synergistic CPT [16]. Pan et al.'s group reported a biodegradable PRDCuS@AG nanocomposite to load doxorubicin and copper sulfide (CuS) for deep tumor CPT [17]. The phototherapy mainly utilizes the NIR light in the wavelength range from 700 to 1100 nm which can efficiently penetrate into biological tissues and selectively act on tumor for an irreversible cellular damage [11]. The chemotherapy, more like the traditional anticancer drug delivery, can be used to kill the cancer cells at the biomolecular level [18].

IR-780 iodide (IR780) photosensitizer is a popular NIR PTT or PDT agent for cancer therapy which holds the suitable fluorescence, good stability, strong absorption in the NIR region, and efficient light conversion [19–21]. Moreover, unlike some synergistic PDT/PTT systems that generally need two lasers with different wavelengths, IR780 dyes can simultaneously produce singlet oxygen and generate heat under one laser irradiation (808 nm), which can avoid the sequential irradiation and shorten treatment time [22, 23]. However, the poor aqueous solubility and concentration-dependent aggregation of IR780 monomers will largely compromise its therapeutic efficacy and should be addressed before their use in biomedical applications [24]. To extend the therapeutic window for IR780, many nanoparticles (NPs), including gold nanostructures [23], carbon nanomaterials [25], and silica NPs [26], were designed to arrange IR780 molecules for PDT and PTT. Compared to others, IR780-doped nanomicelles or self-assembling NPs exhibit advantages in phototherapy [27]. Importantly, these NPs are fabricated using biodegradable polymers, such as poly(D,L-lactide-co-glycolide) (PLGA), which have been extensively studied for many years as therapeutic platforms [28]. Although the strategies of PS-doped nanoplatform have been widely reported, the main challenge in the design of IR780-doped NPs is to confine the PSs at a high concentration inside NPs without a self-quenching because IR780 PSs tend to stack to form H-aggregates in polar solvents such as phosphate-buffered saline (PBS) buffer [29]. Unfortunately, all previously developed carries including self-assembled NPs are limited to precisely control the arrangement and ordering of PSs at a high concentration [30]. Therefore, the key to design highly fluorescent PDT/PTT NPs is to precisely place and align IR780 PSs within crystalline nanomaterials with programmable interfluorophore distance and orientation.

We recently developed a new type of highly stable and dynamic single-walled nanotubes from sequence-defined peptoids (poly-N-substituted glycines) [31]. We have showed that these nanotubes can be programmed for various applications, such as water decontamination and cellular imaging. By taking advantage of easy synthesis of peptoids and robustness of nanotube self-assembly, we have demonstrated the introduction of various functional groups, including fluorescent dye, macrocyclic compound, biomolecule, and peptides, as peptoid side chains within peptoid nanotubes. In addition, because peptoids are highly stable and biocompatible and exhibit protein-like high selectivity [32], these peptoid nanotubes are expected to provide an effective platform for precisely engineering IR780 dyes with well-controlled interfluorophore distance and orientation, developing biocompatible NIR nanomaterials for CPT applications [33–35].

**Scheme 1:**
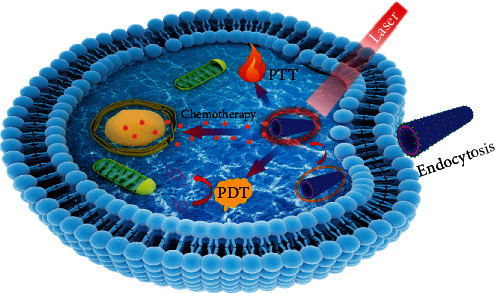
Schematic illustration of DOX-loaded sc-PepIR-40 nanotubes for trimodal chemo-PDT/PTT synergistic therapy of glioma cancer cells, in which the loaded IR780 dyes can simultaneously produce singlet oxygen and generate heat under 808 nm laser irradiation to facilitate the effective PDT/PTT against tumor cells. Furthermore, DOX drug molecules were loaded within PepIR nanotubes for a synergistic chemotherapy treatment.

Malignant gliomas are the most common brain tumors that are highly aggressive and show very high morbidity and mortality [36–38]. They exhibit invasive growth into surrounding normal brain tissues. Chemotherapy remains the main treatment, but the single therapy result is very limited due to its inability to address the highly invasive nature of gliomas [39]. Recently, phototherapy showed the great potential for malignant glioma therapy by using a laser to selectively cause tumor cell apoptosis [40, 41]. For the above reasons, herein, we developed a simple and effective multimodal therapeutic system against gliomas by using the CPT strategy, in which highly stable and crystalline peptoid nanotubes were used as the biocompatible scaffold to precisely display and align IR780 PSs and to simultaneously load chemotherapeutic drug doxorubicin (DOX). Owing to the precise adjustment of IR780 intermolecular distance as a result of nanotube high crystallinity, nanotubes assembled from IR780-conjugated peptoids (PepIR) exhibited a simple, safe, and effective platform for simultaneous PDT/PTT, in which DOX drug molecules were loaded within PepIR nanotubes for a synergistic treatment. The combined CPT strategy (Scheme 1) resulted in significantly higher therapeutic efficiency than individual phototherapy or chemotherapy did.

## 2. Results and Discussion

### 2.1. Synthesis, Assembly, and Characterization of PepIR Nanotubes

**Figure 1:**
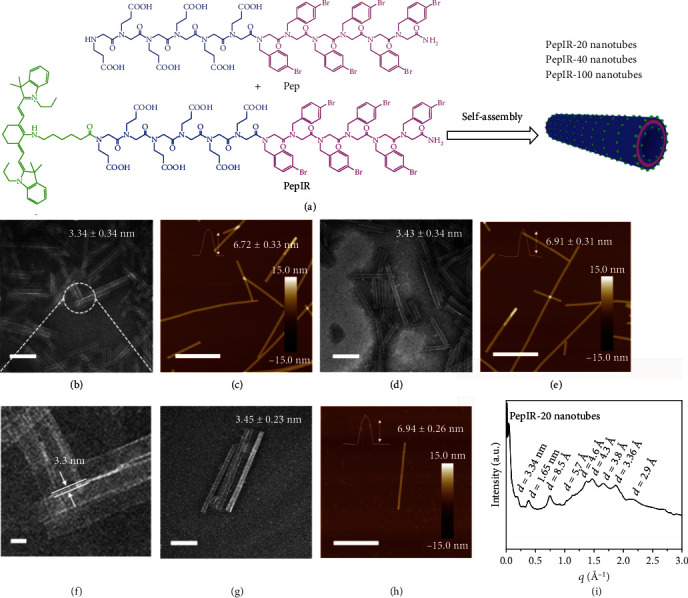
Characterizations of PepIR nanotubes: (a) structures of Pep and PepIR and the scheme showing the self- or coassembly into PepIR nanotubes with a tunable density of IR780 dyes. (b) TEM image of PepIR-20 nanotubes coassembled from 20% PepIR and 80% Pep (scale bar, 200 nm). (c) *Ex situ* AFM image of PepIR-20 nanotubes (scale bar, 1.0 *μ*m). The top inset is the height of PepIR-20 nanotube. 20 nanotubes were analyzed for obtaining the height distribution. (d) TEM image of PepIR-40 nanotubes coassembled from 40% PepIR and 60% Pep (scale bar, 200 nm). (e) *Ex situ* AFM image of PepIR-40 nanotubes (scale bar, 1.0 *μ*m). The inset is the height of PepIR-40 nanotube. 20 nanotubes were analyzed for obtaining the height distribution. (f) The high-resolution TEM image showing the wall thickness of nanotubes in (b) (scale bar, 10 nm). (g) TEM image of PepIR-100 nanotubes assembled from 100% PepIR (scale bar, 200 nm). (h) *Ex situ* AFM image of PepIR-100 nanotubes (scale bar, 1.0 *μ*m). The inset is the height of PepIR-100 nanotube. 20 nanotubes were analyzed for obtaining the height distribution. (i) XRD spectrum of PepIR-20 nanotubes. The formula of *d* = 2*π*/*q* was used to calculate the values above each peak.

According to a previously developed solid-phase submonomer synthesis method [31], tube-forming peptoids with and without IR780 were designed and synthesized. As shown in Figure 1(a), these peptoid sequences have six N-(2-carboxyethyl) glycine (Nce) groups as the polar domain and six N-[(4-bromophenyl)methyl] glycine (Nbrpm) groups as the hydrophobic region. The IR780 PS was conjugated at the N-terminus adjacent to the polar domain. The detailed preparation, purification, and characterizations of these two peptoids (Pep: Nbrpm_6_Nce_6_; PepIR: Nbrpm_6_Nce_6_Nc_6_IR780) are described in the Materials and Methods and supporting information (Figure S1). Peptoid nanotubes with a tunable density of IR780 PSs were synthesized by coassembling PepIR with Pep in a variable ratio using a similar evaporation-induced crystallization approach described previously (see Materials and Methods) [31]. As shown in Figure 1, both transmission electron microscopy (TEM) and atomic force microscopy (AFM) data showed that nanotubes assembled from 100% PepIR (PepIR-100), 40% PepIR (PepIR-40), or 20% PepIR (PepIR-20) all have similar structure. TEM images showed that PepIR-20 nanotubes exhibited a diameter of 32.66 ± 1.90 nm (Figure S3a) and a wall thickness of 3.34 ± 0.34 nm (Figure 1(b)). A high-resolution image of these nanotubes is shown in Figure 1(f), and the increased tube wall thickness is due to the introduction of IR780 PSs within peptoid nanotubes [31]. TEM results showed that PepIR-40 nanotubes exhibited a wall thickness of 3.43 ± 0.34 nm (Figure 1(d) and S2c) and a diameter of 33.71 ± 2.7 nm (Figure S3b), and PepIR-100 nanotubes had a wall thickness of 3.45 ± 0.23 nm (Figure 1(g) and S2e) and a diameter of 35.67 ± 2.04 nm (Figure S3c). The heights of PepIR-20, PepIR-40, and PepIR-100 nanotubes from ex situ AFM results are 6.72 ± 0.33 nm, 6.91 ± 0.31 nm, and 6.94 ± 0.26 nm, respectively (Figures 1(c), 1(e), and 1(h)), which are approximately two times the wall thickness, showing the deformation of PepIR nanotubes under dry conditions [26]. The X-ray diffraction (XRD) data proved that these PepIR nanotubes have crystalline nanostructures and PepIR-20, PepIR-40, and PepIR-100 nanotubes all exhibited similar structures by having similar XRD patterns (Figure 1(i), Figure S4a and b). As shown in Figure 1(i), the first low *q* peak (*d* = 3.34 nm) indicates the wall thickness of PepIR-20 nanotube, which is consistent with the data analyzed by TEM tests (Figures 1(b) and 1(f)). The peak at 1.65 nm is the distance between two peptoid backbones in the direction of the hydrophobic Nbrpm groups facing each other [31]. The spacing of 5.7 Å is attributed to the ordered packing of aromatic side chains Nbrpm_6_. The 4.6 Å spacing corresponds to the alignment of lipid-like peptoid chains. The peak at 3.36 Å indicates the interresidue distance along the chain direction. And the peaks at 4.3 Å, 3.8 Å, and 2.9 Å indicate the presence of extensive *π*-stacking. As the model we proposed in previous work [31], the hydrophobic Nbrpm groups are packed with each other and embedded in the center of the nanotube wall. As for the polar Nce groups, they are located on both surfaces of the nanotube and exhibit a similar packing to Pep nanotubes that we reported previously [31]. These results showed that IR780 dye molecules could be precisely displayed within crystalline nanotubes with a tunable density while retaining the similar tubular framework structure.

**Figure 2:**
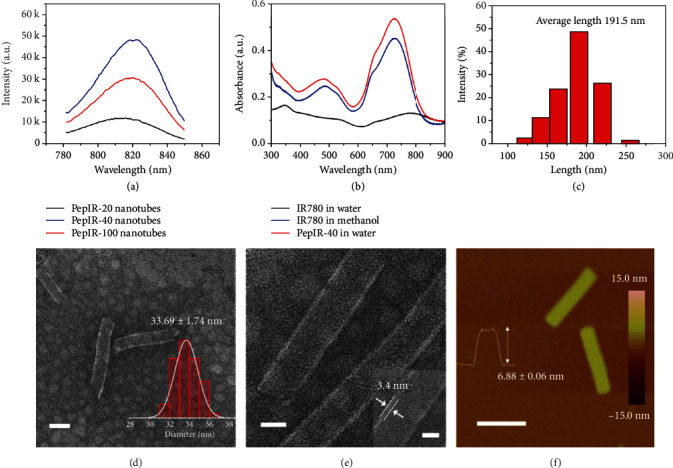
(a) Fluorescence spectra of PepIR-20, PepIR-40, and PepIR-100 nanotubes. (b) UV-vis data of IR780 dye in water/methanol and PepIR-40 nanotubes in water. (c) Dynamic light scattering (DLS) data of sc-PepIR-40 nanotubes showing that the average tube length is about 191.5 nm. (d) TEM image of sc-PepIR-40 nanotubes (scale bar, 50 nm). The right inset is the statistical tube diameter distribution measured from the TEM results. The average tubular diameter is shown above the histogram. (e) Magnified TEM image of sc-PepIR-40 nanotubes (scale bar, 20 nm). The right inset is the high-resolution TEM image showing the tube wall thickness of 3.4 nm (scale bar, 10 nm). (f) *Ex situ* AFM image of two sc-PepIR-40 nanotubes (scale bar, 200 nm). The inset is the AFM height measurement showing the sc-PepIR-40 nanotube height. 20 nanotubes were analyzed for obtaining the height distribution.

The main challenge in the design of PS-doped nanoparticles for phototherapy is to address the issue of self-quenching of the PSs [27, 42]. Indeed, as flat aromatic structures, IR780 PSs tend to form *π*-stacking aggregates (H-aggregates) that exhibit poor fluorescent signals [29]. Crystalline nanotubes exhibit well-defined orientations and distances of functional groups [31], which is an obvious advantage for engineering IR780 PSs at a high concentration for phototherapy. To demonstrate that, we analyzed the fluorescence properties of these PepIR-20, PepIR-40, and PepIR-100 nanotubes. As shown in Figure 2(a), PepIR nanotubes gradually increased their fluorescence emission intensity as the percentage of PepIR increased from 20% to 40%, while the fluorescence emission intensity of PepIR-100 nanotubes significantly decreased in contrast to the PepIR-40 nanotubes which might be due to the self-quenching of some IR780 dyes. These results indicated that the PepIR-40 nanotube is the best candidate for the phototherapy among these three types of nanotubes. Besides, the UV-vis absorption of PepIR-40 nanotubes in water is significantly higher than those free IR780 PSs in methanol or water (Figure 2(b)), which further indicated that the high crystallinity of peptoid nanotubes facilitated the fluorescence emission intensity of IR780 PSs by reducing the self-quenching effect in aqueous solution. These PepIR-40 nanotubes are good candidates for biological applications. Furthermore, the high loading molar ratio of IR780 PSs within PepIR-40 nanotubes indicates that peptoid-based coassembly strategy is an effective way for precisely controlling the loading and ordering of PSs at high concentrations.

### 2.2. Synthesis and Characterization of Colloidal PepIR-40 Nanotubes

To improve cellular uptake of PepIR-40 nanotubes, the prepared nanotubes were sonication-cut (see Materials and Methods section), and the dynamic light scattering (DLS) data showed that the obtained colloidal PepIR-40 nanotubes (denoted as sc-PepIR-40 nanotubes) have an average of length of ~191.5 nm (Figure 2(c)). TEM images (Figure 2(d), Figure S5a and b) showed that these sc-PepIR-40 nanotubes retained the tubular morphology with a diameter of ~33.69 ± 1.74 nm (inset in Figure 2(d)), which is consistent with that of presonicated PepIR-40 nanotubes. Moreover, as shown in the high-resolution TEM image in Figure 2(e), the wall thickness of these sonication-cut nanotubes (3.4 nm) is also comparable to presonicated PepIR-40 nanotubes. The height measured from ex situ AFM image of sonication-cut nanotubes is around 6.88 ± 0.06 nm (Figure 2(f)), similar to the height obtained from presonicated PepIR-40 nanotubes (Figure 1(e)). All these results indicate that sonication is an effective way to cut PepIR nanotubes into a short length without changing other structural parameters.

### 2.3. The Photothermal Behavior and ^1^O_2_ Generation of sc-PepIR-40 Nanotubes

**Figure 3:**
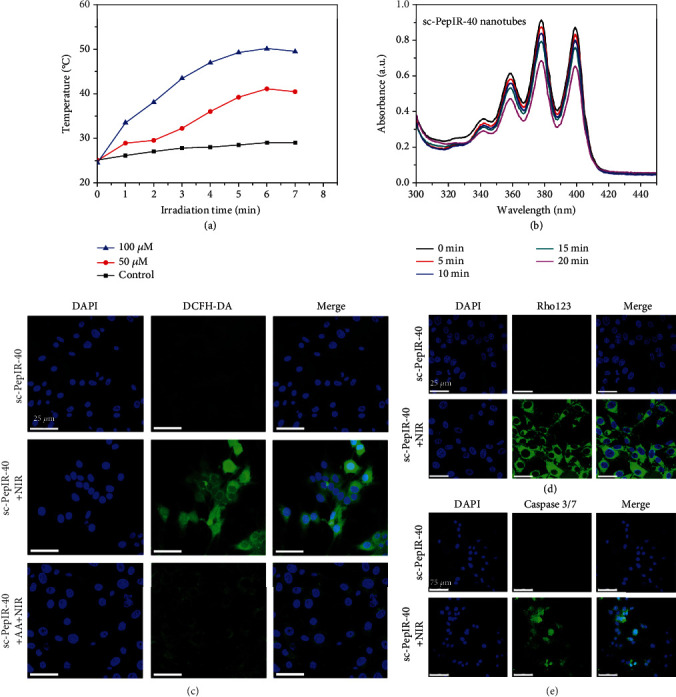
(a) Photothermal effect of different concentrations (50 *μ*M and 100 *μ*M) of sc-PepIR-40 nanotubes under 808 nm laser irradiation, and PBS buffer was used as the control. (b) UV-vis absorption spectra of ABDA under 808 nm laser irradiation for different times in the presence of sc-PepIR-40 nanotubes. Intracellular ^1^O_2_ generation of sc-PepIR-40 nanotubes in U87MG cells with/without NIR laser irradiation detected by DCFH-DA (c), Rho123 (d), and caspase 3/7 kit (e). Cell nuclei were stained with DAPI in blue color. All laser irradiation was 5 min (808 nm, 1.8 W cm^−2^).

After the sonication cutting and characterizations of these PepIR nanotubes, we then studied their photothermal behaviors (Figure 3(a)). The temperature of the nanotube aqueous solution increased to 49.2°C in 5 min under the 808 nm laser irradiation, and the maximum temperature was 50.1°C. It has been reported that hyperthermia can kill tumor cells directly at >45°C [14], and the above temperatures are high enough to cause significant hyperthermia damage to cancer cells while peptoid nanotubes are stable in such condition (Figure S6). These results showed that sc-PepIR-40 nanotubes proved the sufficient photothermal conversion capability suitable for a PTT application.

To verify the photodynamic effects of these sc-PepIR-40 nanotubes, they were evaluated for ^1^O_2_ generation using 9,10-anthracenediyl-bis (methylene) dimalonic acid (ABDA) as an indicator. ABDA could undergo oxidation to yield an endoperoxide once ^1^O_2_ exist, resulting in the decreased absorption of ABDA. As shown in Figure S7, in the absence of sc-PepIR-40 nanotubes, there was no obvious decrease of ABDA absorption under 808 nm laser irradiation, while after adding sc-PepIR-40 nanotubes, the ABDA absorption was gradually decreased under 808 nm laser irradiation (Figure 3(b)), proving that ^1^O_2_ was generated and sc-PepIR-40 nanotubes can be used for cancer PDT.

To investigate whether sc-PepIR-40 nanotubes could be used for effective intracellular PDT, 2′,7′-dichlorofluorescin diacetate (DCFH-DA) was used along with sc-PepIR-40 nanotubes to detect intracellular ^1^O_2_ generation of these nanotubes under NIR laser (Figure 3(c)). After cellular uptake, DCFH-DA is converted to nonfluorescent 2′,7′-dichlorodihydrofluorescein (DCFH), and the nanotube-induced ^1^O_2_ generation can oxidize DCFH to generate green fluorescence. As shown in Figure 3(c), a fluorescence signal was barely observed in the U87MG cells treated with sc-PepIR-40 nanotubes without 808 nm laser irradiation. In contrast, the nanotube-treated cells under 808 nm laser irradiation exhibited a strong green fluorescence signal, suggesting that ^1^O_2_ were generated from sc-PepIR-40 nanotubes within U87MG cells upon NIR laser irradiation. When the ^1^O_2_ scavenger ascorbic acid (AA) was premixed with sc-PepIR-40 nanotubes before laser irradiation, the green fluorescence signal significantly decreased, which indicated that the appearance of green fluorescence signal was a result of ^1^O_2_ generation [43]. In addition, a burst of ^1^O_2_ can disrupt the normal physiological redox state, resulting in the destruction of mitochondrial membrane potential (MMP), as indicated by using rhodamine 123 (Rho 123) [44, 45]. As shown in Figure 3(d), compared to the control group without NIR laser irradiation, a bright green fluorescence signal was observed within U87MG cells when they were incubated with sc-PepIR-40 nanotubes for 4 h and treated with 808 nm laser irradiation, indicating the destruction of mitochondria. These results showed that sc-PepIR-40 nanotubes under 808 nm laser irradiation could generate ^1^O_2_ within U87MG cells. PDT-induced apoptosis of cancer cells can be analyzed using a caspase 3/7 kit, a novel fluorogenic substrate for activated caspase-3/7, which is an essential event during apoptosis [46]. As shown in Figure 3(e), a treatment of U87MG cells with sc-PepIR-40 nanotubes without 808 nm laser irradiation showed no effect on caspase activity, while an obvious green fluorescence signal of activated caspase 3/7 was observed when these cells were incubated with sc-PepIR-40 nanotubes under 808 nm laser irradiation, further confirming that sc-PepIR-40 nanotubes under 808 laser irradiation can induce effective cell apoptosis and be used for PDT.

### 2.4. Drug Loading, Release, and Cellular Uptake of sc-PepIR-40 Nanotubes

**Figure 4:**
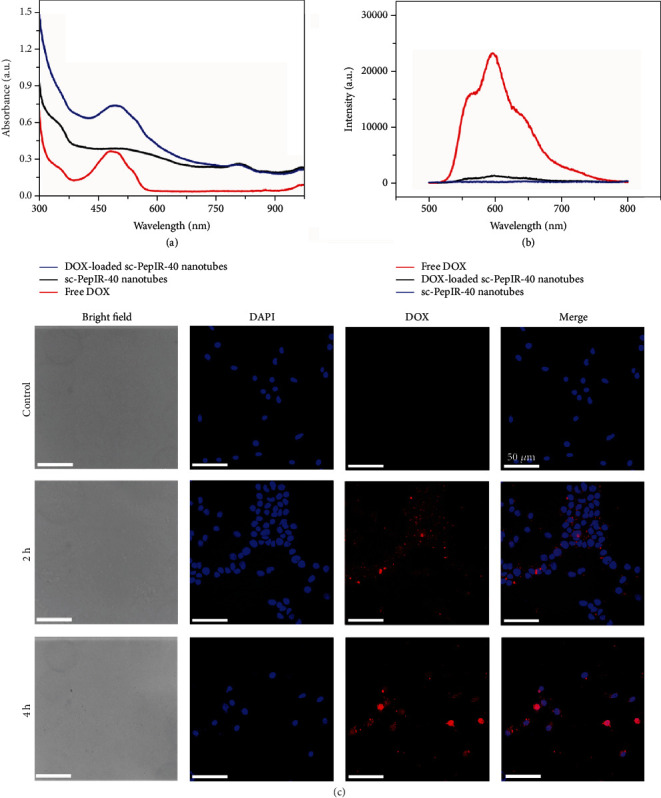
UV-vis absorbance spectra (a) and fluorescence spectra (b) of sc-PepIR-40 nanotubes, free DOX, and DOX-loaded sc-PepIR-40 nanotubes. (c) CLSM images of U87MG cells incubated with DOX-loaded sc-PepIR-40 nanotubes for 2 h and 4 h. Blue color comes from cell nuclei stained with DAPI.

DOX, a well-known anticancer drug, was chosen as a model drug that was loaded in these sc-PepIR-40 nanotubes with a loading content up to ~27.6% (calculated by the loading experiments described in Materials and Methods section). While the UV-vis spectra of the sc-PepIR-40 nanotubes showed the characteristic peak of IR780 at ∼800 nm (Figure 4(a)), the DOX-loaded sc-PepIR-40 nanotubes had the characteristic peaks of both DOX and IR780 in the UV-vis spectra (Figure 4(a)). The successful loading of DOX within sc-PepIR-40 nanotubes was further confirmed by the fluorescent measurements (Figure 4(b)). Compared to that of free DOX, the fluorescence emission of DOX-loaded sc-PepIR-40 nanotubes was significantly quenched. The cellular uptake and drug release behavior of the DOX-loaded sc-PepIR-40 nanotubes in U87MG cells were analyzed by confocal laser scanning microscopy (CLSM). The fluorescence emission of DOX was quenched after being loaded in the sc-PepIR-40 nanotubes. However, the recovery of DOX fluorescence within cancer cells was observed when the DOX was released from nanotubes. Therefore, we used the red fluorescence signal of DOX to track the intracellular drug release (Figure 4(c)). After 2 h of incubation, compared with the cells in the control group, the glioma cancer cells treated with DOX-loaded sc-PepIR-40 nanotubes showed an obvious red color in the cytoplasm. Furthermore, DOX was gradually distributed in the nuclear area after 4 h of incubation. These results indicated that DOX was successfully released from sc-PepIR-40 nanotubes within glioma cancer cells.

### 2.5. In Vitro Cytotoxicity Assay of sc-PepIR-40 Nanotube-Based System

**Figure 5:**
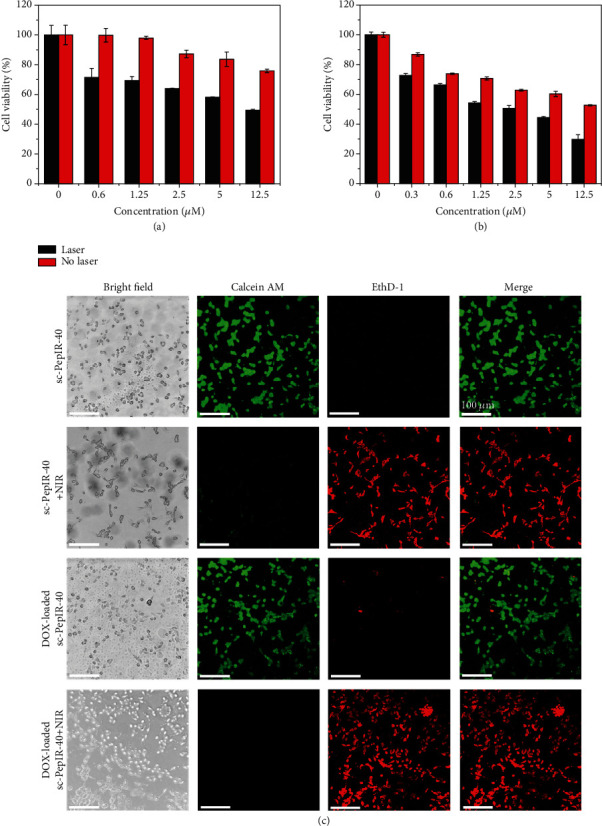
MTT assay of U87MG cells incubated with sc-PepIR-40 nanotubes with/without 808 nm laser irradiation (a) and DOX-loaded sc-PepIR-40 nanotubes with/without 808 nm laser irradiation (b). (c) CLSM images of cell viability assay after incubating with sc-PepIR-40 nanotubes or with DOX-loaded sc-PepIR-40 nanotubes with/without NIR laser irradiation. The cells were stained with live/dead viability kit (live cells were labeled with calcein AM with green fluorescence, and the dead cells were shown in red fluorescence after being labeled with EthD-1). All laser irradiations were performed using an 808 nm laser with a power intensity of 1.8 W cm^−2^ for 5 minutes.

The synergistic effect of DOX-loaded sc-PepIR-40 nanotubes on the apoptosis of U87MG cells was investigated by the (3-(4,5-dimethylthiazol-2-yl)-2,5-diphenyltetrazolium bromide) (MTT) assay.  Figure 5 described the MTT data; sc-PepIR-40 nanotubes without (Figure 5(a)) and with a loading of DOX (Figure 5(b)) both exhibited an increased cytotoxicity in a concentration-dependent manner after 48 h incubation under 808 nm laser irradiation. For the DOX-loaded sc-PepIR-40 nanotubes treated with laser irradiation (808 nm), over 70% of the cells were killed at peptoid tube concentration of 12.5 *μ*M, indicating that this combined treatment had a significantly high therapeutic efficiency against glioma cell growth. Moreover, the half-maximal inhibitory concentrations (IC_50_) of these treatments are shown in Table S1, and the combination index (CI) was calculated to be 0.323 (<1), confirming a high synergistic therapeutic effect [47].

To visually estimate the in vitro therapeutic effect of these sc-PepIR-40 nanotubes, we used a live/dead cell viability assay kit to evaluate the influence of the combined treatment in killing glioma cells. Live cells were labeled with calcein AM with green fluorescence, and the dead cells were shown in red fluorescence after being labeled with ethidium homodimer-1 (EthD-1).  Figure 5(c) showed the confocal images of the live/dead cells marked with calcein-AM and EthD-1 after incubating U87MG cells with sc-PepIR-40 nanotubes or DOX-loaded sc-PepIR-40 nanotubes for 4 h, respectively. Before 808 nm laser irradiation, while DOX-loaded nanotubes induced an obvious cell death, almost no dead cells were induced by sc-PepIR-40 nanotubes. Interestingly, upon 808 nm laser irradiation, even sc-PepIR-40 nanotubes induced a significant population of dead cells (Figure 5(c)), while DOX-loaded sc-PepIR-40 nanotubes induced a much higher population of dead cells as sc-PepIR-40 nanotubes did. These results further confirmed the potent phototherapeutic efficacy of sc-PepIR-40 nanotubes and the synergistic effect of DOX-loaded sc-PepIR-40 nanotubes on the treatment of malignant glioma as a result of simultaneous chemo-PDT/PTT polytherapy. This result is also consistent with the conclusion obtained from the MTT assay above (Figures 5(a) and 5(b)).

## 3. Conclusion

In summary, PS-doped peptoid-based crystalline nanotubes, in which IR780 was attached at the N-terminus of tube-forming peptoid sequences, were coassembled and developed as an efficient platform to deliver DOX for the simultaneous chemo-PDT/PTT trimodal treatment of glioma. In this versatile system, the IR780 PSs were effectively packed and separated from each other within crystalline nanotubes; thus, a self-quenching of these IR780 molecules was significantly reduced. The precise placement of IR780 dyes within peptoid nanotubes enabled the high stability of IR780 for an excellent ^1^O_2_ production and photothermal conversion within glioma cancer cells. Moreover, efficient DOX-loading was achieved because of the large surface area of the nanotubes, contributing to an efficient CPT strategy against the glioma cells. The enhanced antitumor efficiency of DOX-loaded sc-PepIR-40 nanotubes was confirmed by the CLSM and MTT assays. Given the above results and the unique properties of peptoids and peptoid nanotubes, the developed multimodal DOX-loaded sc-PepIR-40 nanotubes in this work offer great promises for future glioma therapy in clinic.

## 4. Materials and Methods

### 4.1. Methods for Synthesizing Pep (Nbrpm_6_Nce_6_) Using a Solid-Phase Synthesizer

The relative peptoids were synthesized on a commercial Aapptec Apex 396 robotic synthesizer using a solid-phase submonomer cycle according to a previously developed solid-phase submonomer synthesis method [31].

### 4.2. Synthesis of Aminohexanoic Acid-Modified Peptoids (PepNc_6_, Nbrpm_6_Nce_6_Nc_6_)

The resins containing Nbrpm_6_Nce_6_Nc_6_ were obtained through the previously reported method [31]. Briefly, the Nc_6_ group was conjugated on the peptoid manually. Then, the resin containing Nbrpm_6_Nce_6_ obtained from automated solid-phase synthesis was mixed with a DMF solution of Fmoc-6-aminohexanoic acid (1.5 mL, 0.9 mmol) and 0.50 mL of 50% (*v*/*v*) DIC/DMF. After agitating the mixture at room temperature for overnight, the resin was filtered and washed with DMF. The terminal Fmoc group was removed by an addition of 2 mL of 20% (*v*/*v*) 4-methylpiperidine/DMF. The mixture was agitated for 1 h; then, the resin was filtered and washed well with DMF.

### 4.3. Synthesis of IR780-Modified Peptoids (PepIR, Nbrpm_6_Nce_6_Nc_6_IR780)

The resins (0.09 mmol) containing PepNc_6_ were mixed with 6.0 mL CH_3_CN solution of IR780 dye (0.9 mmol) and DIPEA (diisopropylethylamine, 0.99 mmol). The mixture was agitated for 24 hours at 80°C (reflux), filtered, and washed well with CH_3_CN and DMF. The resins containing PepIR were synthesized.

### 4.4. Peptoid Cleavage and HPLC Purification

The final crude products (Pep or PepIR) were cleaved from the corresponding resins by using 95% trifluoroacetic acid (TFA) in water, which was conducted following the previously reported method [31]. Finally, the peptoid powder was stored at -80°C.

### 4.5. Assembly of PepIR Nanotubes

Pep and PepIR were coassembled in the mixture of H_2_O/CH_3_CN (*v*/*v* = 3 : 7). For coassembly, peptoids in a relative ratio were dissolved in the mixture of H_2_O and CH_3_CN (*v*/*v* = 3 : 7) with peptoid concentration which was 5.0 mM. Then, the solutions were put in a refrigerator at 4°C to achieve slow evaporation. After 48 h, the cloudy solution containing crystalline nanotubes was prepared.

### 4.6. Synthesis of sc-PepIR-40 Nanotubes by Sonication

The cloudy aqueous solution that contains diluted peptoid nanotubes (250 *μ*M) was sonicated using an ultrasonic processor (FS-450N, 150 W) for 5 min; ice water was used to keep the nanotube solution temperature consistent during the sonication-cutting process.

### 4.7. Photothermal Heating Effect of Sonicated sc-PepIR-40 Nanotubes

Different concentrations of sc-PepIR-40 nanotubes (50 *μ*M and 100 *μ*M) were treated under 808 nm laser irradiation. The PBS solution containing no sc-PepIR-40 nanotubes was also investigated as the control. The temperatures at different time points were recorded using a thermometer.

### 4.8. Loading DOX to sc-PepIR-40 Nanotubes

150 *μ*L aqueous solution of DOX (1.0 mg/mL) was mixed with 800 *μ*L aqueous solution of sc-PepIR-40 nanotubes (250 *μ*M), and the resulting mixture was stirred under the dark condition for 24 h. After removing all unloaded DOX through centrifugation, the obtained DOX-loaded sc-PepIR-40 nanotubes were redispersed in 800 *μ*L H_2_O and diluted to the corresponding assay by checking the UV-vis absorbance at 490 nm. Based on a calibration curve obtained from four DOX solutions at different concentrations (Figure S8), the amount of loaded DOX within sc-PepIR-40 nanotubes was determined. The drug loading content was determined by the following equation:

(1) $$\begin{array}{c} \text{Drug loading content }\left(\%\right)=\frac{\text{weight of drug in nanotubes}}{\text{weight of nanotubes taken}}\times 100. \end{array}$$

### 4.9. Cellular Uptake of DOX-Loaded sc-PepIR-40 Nanotubes

U87MG cells were incubated in DMEM and seeded in a 12-well culture plate for 24 h. After washing with PBS, the cells were incubated with DOX-loaded sc-PepIR-40 nanotubes (2.5 *μ*M) for 2 h and 4 h, respectively. Cells without nanotube treatment were used as the control. 4% paraformaldehyde was used to fix cells, and cell nuclei were stained with DAPI in blue color for CLSM observation (excitation at 405 nm and 488 nm).

### 4.10. Detection of ^1^O_2_ Generation

The extracellular ^1^O_2_ generation was monitored with ABDA, a ^1^O_2_ monitor *via* its decreased fluorescence absorption due to the reaction with ^1^O_2_. In the experiment, 400 *μ*M ABDA with and without sc-PepIR-40 nanotubes (25 *μ*M) were under the 808 nm laser irradiation (1.8 W cm^−2^). Their ABDA absorbance changes at different irradiation time intervals (0-20 min) were tested using a microplate reader.

The intracellular ^1^O_2_ generation was monitored with DCFH-DA, a probe that turned to highly green fluorescent 2′,7′-dichlorofluorescein in the presence of ^1^O_2_. U87MG cells were seeded in a 12-well culture plate for 24 h and incubated 4 h with sc-PepIR-40 nanotubes. Then, 1.0 mL DMEM containing 10 *μ*M DCFH-DA was added to each well with incubation at 37°C for 20 min. In addition, 10 *μ*M DCFH − DA + 100 *μ*M AA were added as the control. Cells without the laser irradiation were also added as the control. After washing with PBS, the cells were irradiated by 808 nm laser (1.8 W cm^−2^, 5 min) and cell nuclei were stained with DAPI for CLSM observation.

The rupture of mitochondrial membrane potential (MMP) and mitochondrial-mediated apoptosis were measured using rhodamine 123 (Rho 123) and caspase 3/7 kit, respectively. In these experiments, U87MG cells were seeded in the culture dishes at a density of 1 × 10^5^ cells per dish. After 24 h incubation at 37°C, cells were incubated with sc-PepIR-40 nanotubes for 4 h and treated with a 5 min laser irradiation (808 nm, 1.8 W cm^−2^). After a further 4 h incubation, Rho 123 (10 *μ*M, 30 min) and caspase 3/7 kits (2 drops/mL, 30 min) were used to stain cells, respectively. CLSM observation was conducted to obtain fluorescence images.

### 4.11. Therapy Efficacy

U87MG cells were seeded in a 12-well culture plate for 24 h and incubated with sc-PepIR-40 nanotubes and DOX-loaded sc-PepIR-40 nanotubes for 4 h. Then, a 5 min laser irradiation (808 nm, 1.8 W cm^−2^) was conducted. As a control, cells were incubated with these nanotubes for 4 h without a laser irradiation. After that, calcein AM (2.0 *μ*M) and EthD-1 (4.0 *μ*M) solutions were added to each well to costain cells at 37°C for 15 min. Finally, CLSM observations were conducted to visualize assay cell viability. To investigate cell viability by MTT assay, the incubation time between U87MG cells and samples was extended to 24 h before the irradiation by 808 nm laser (1.8 W cm^−2^, 5 min). Then, the cells were further incubated for 24 h and the viability was detected by a microplate reader. Each well was obtained by following the MTT kit instruction.

## Data Availability

All data are available in the manuscript, supplementary materials, or from the author.

## Supplementary Materials

Supplementary Materials Figure S1: ultraperformance liquid chromatography (UPLC) and mass spectrometry (MS) data of peptoids: (a) Nbrpm6Nce6 (Pep) and (b) Nbrpm6Nce6Nc6IR780 (PepIR). Figure S2: (a) TEM image of PepIR-20 nanotubes (scale bar, 500 nm). (b) Ex situ AFM image of PepIR-20 nanotubes (scale bar, 2.0 *μ*m). (c) TEM image of PepIR-40 nanotubes (scale bar, 500 nm). (d) Ex situ AFM image of PepIR-40 nanotubes (scale bar, 2.0 *μ*m). (e) TEM image of PepIR-100 nanotubes (scale bar, 200 nm). Figure S3: statistical diameter distribution of PepIR-20 nanotubes (a), PepIR-40 nanotubes (b), and PepIR-100 nanotubes (c) measured from the TEM results. The number above the histogram is the average tubular diameter; 50 nanotubes were analyzed for each size distribution. Figure S4: XRD data of PepIR-40 nanotubes (a) and PepIR-100 nanotubes (b). The values above each peak were calculated according to the formula of *d* = 2*π*/*q*, and these numbers are similar to those of Pep nanotubes, indicating that PepIR-20, PepIR-40, and PepIR-100 nanotubes all exhibited similar structures to those of Pep nanotubes. Figure S5: TEM images ((a) scale bar, 50 nm; (b) scale bar, 100 nm) and ex situ AFM image ((c) scale bar, 500 nm) of sc-PepIR-40 nanotubes as a result of ultrasonication. Figure S6: TEM images ((a, b) scale bar, 50 nm; (c) scale bar, 20 nm) and ex situ AFM images ((d) scale bar, 200 nm; (e, f) scale bar, 500 nm) of sc-PepIR-40 nanotubes after being treated at 50°C. These results showed the high stability of these sc-PepIR-40 nanotubes, which remained to have the same tubular morphology after being incubated in aqueous solution at 50°C for 6 h. Figure S7: UV-vis absorption spectra of ABDA under 808 nm laser irradiation for different times. There was no obvious change in ABDA absorption after exposure to 808 nm laser irradiation. Figure S8: the calibration curve obtained from four DOX solutions at different concentrations. Table S1: the half-maximal inhibitory concentration (IC50) (*μ*M) of U87MG cells under different treatments. The first row shows the different treatments of U87MG cells; the second row shows the targets that we calculated IC50; the third row shows the values of the corresponding IC50 (*μ*M). The IC50 of DOX in chemotherapy and the IC50 of sc-PepIR-40 nanotubes in phototherapy are 17.37131 *μ*M and 10.954 *μ*M, respectively. A significant decrease of IC50 in combined therapy was observed (2.1078 *μ*M). Moreover, the combination index (CI) was calculated to be 0.323 (<1), confirming a high synergistic therapeutic effect of chemotherapy and phototherapy.
