# Supplementary material for: Sequence-Defined Nanotubes Assembled from IR780-Conjugated Peptoids for Chemophototherapy of Malignant Glioma
Source: Research (Wash D C). 2021 May 16;2021:9861384. doi: 10.34133/2021/9861384 (PMC8147695; doi:10.34133/2021/9861384)
Supplement: Supplementary Materials — Figure S1: ultraperformance liquid chromatography (UPLC) and mass spectrometry (MS) data of peptoids: (a) Nbrpm6Nce6 (Pep) and (b) Nbrpm6Nce6Nc6IR780 (PepIR). Figure S2: (a) TEM image of PepIR-20 nanotubes (scale bar, 500 nm). (b) Ex situ AFM image of PepIR-20 nanotubes (scale bar, 2.0 μm). (c) TEM image of PepIR-40 nanotubes (scale bar, 500 nm). (d) Ex situ AFM image of PepIR-40 nanotubes (scale bar, 2.0 μm). (e) TEM image of PepIR-100 nanotubes (scale bar, 200 nm). Figure S3: statistical diameter distribution of PepIR-20 nanotubes (a), PepIR-40 nanotubes (b), and PepIR-100 nanotubes (c) measured from the TEM results. The number above the histogram is the average tubular diameter; 50 nanotubes were analyzed for each size distribution. Figure S4: XRD data of PepIR-40 nanotubes (a) and PepIR-100 nanotubes (b). The values above each peak were calculated according to the formula of d = 2π/q, and these numbers are similar to those of Pep nanotubes, indicating that PepIR-20, PepIR-40, and PepIR-100 nanotubes all exhibited similar structures to those of Pep nanotubes. Figure S5: TEM images ((a) scale bar, 50 nm; (b) scale bar, 100 nm) and ex situ AFM image ((c) scale bar, 500 nm) of sc-PepIR-40 nanotubes as a result of ultrasonication. Figure S6: TEM images ((a, b) scale bar, 50 nm; (c) scale bar, 20 nm) and ex situ AFM images ((d) scale bar, 200 nm; (e, f) scale bar, 500 nm) of sc-PepIR-40 nanotubes after being treated at 50°C. These results showed the high stability of these sc-PepIR-40 nanotubes, which remained to have the same tubular morphology after being incubated in aqueous solution at 50°C for 6 h. Figure S7: UV-vis absorption spectra of ABDA under 808 nm laser irradiation for different times. There was no obvious change in ABDA absorption after exposure to 808 nm laser irradiation. Figure S8: the calibration curve obtained from four DOX solutions at different concentrations. Table S1: the half-maximal inhibitory concentration (IC50) (μM) of U87MG cells under dif [file 9861384.f1.doc]

Supporting Information

**Sequence-Defined Nanotubes Assembled from IR780-Conjugated Peptoids for Chemo-Phototherapy of Malignant Glioma**

Xiaoli Cai,^1,†^ Mingming Wang,^2,†^ Peng Mu,^2,3^ Tengyue Jian,^2^ Dong Liu,^1^ Shichao Ding,^1^ Yanan Luo,^1^ Dan Du,^1^ Yang Song,^1*^ Chun-Long Chen,^2*^ and Yuehe Lin^1*^

^1^ School of Mechanical and Materials Engineering, Washington State University, PO Box 642920 Pullman, Washington 99164, United States

^2^ Physical Sciences Division, Pacific Northwest National Laboratory, Richland, WA 99352, United States

^3^ Department of Mechanical Engineering and Materials Science and Engineering Program, State University of New York, Binghamton, New York 13902, United States

^†^Both authors contributed equally to this manuscript

**Materials and Chemicals.** IR-780 iodide (IR780), doxorubicin hydrochloride (DOX)，dimalonic acid (ABDA) and 2,7-dichlorodihydrofluoresceindiacetate (DCFH-DA), ascorbic acid (AA), 3-(4,5-Dimethylthiazol-2-yl)-2,5-diphenyltetrazolium bromide (MTT), 6-diamidino-2-phenylindole (DAPI), 4% formaldehyde, and dimethyl sulfoxide (DMSO) were all purchased from Sigma-Aldrich (MA, USA). Live/dead
viability kit, rhodamine 123 (Rho 123) and caspase 3/7 kit were obtained
from Thermo Fisher Scientific (USA). Fetal bovine serum (FBS), penicillin and streptomycin for cell cultures were purchased from ATCC. Dulbecco's Modified Eagle's Medium (DMEM) and phosphate buffer saline (PBS) were supplied by Gibco. The U87MG cell lines were ordered from ATCC. Other reagents, if not specified, were purchased from local suppliers. The water in all experiments was prepared in a three-stage Millipore Milli-Q plus 185 purification system and had a resistivity higher than 18.2 MΩ cm.

**Apparatus.** Transmission electron microscopy (TEM) images were obtained using a FEI Tecnai instrument. Atomic force microscopy (AFM) tests were performed on a Bruker MultiMode 8 by using tapping mode or ScanAsyst mode at room temperature. UV-vis spectra and fluorescence spectra were obtained with a Tecan Safire2 microplate reader. Confocal laser scanning microscope (CLSM) studies were observed using a Leica TCS SP8 microscope.

**(a)**


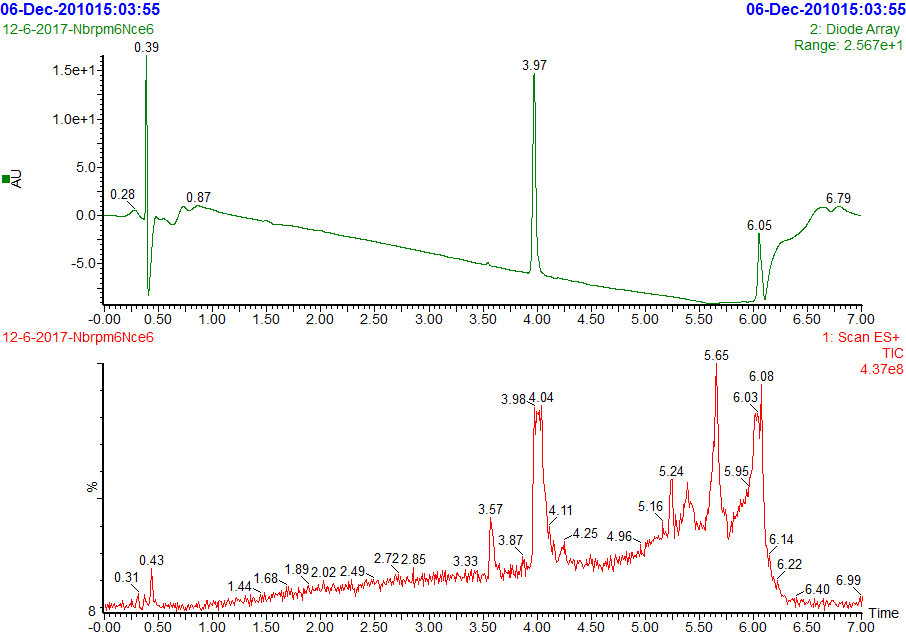


**
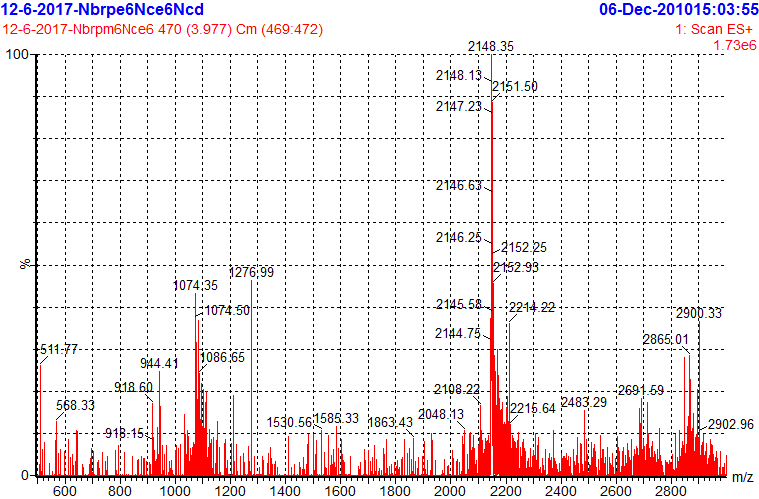
**

**
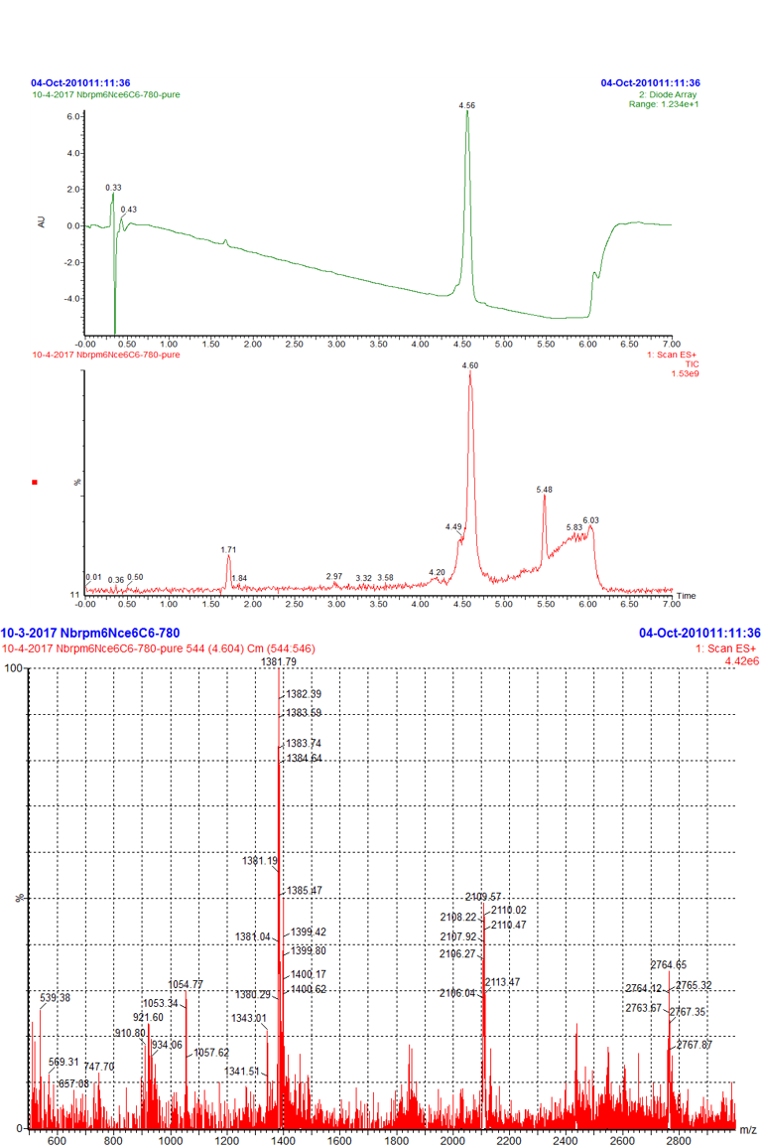
**

**(b)**

**Figure S1.** Ultra-performance liquid chromatography (UPLC) and mass spectrometry (MS) data of peptoids: (a) Nbrpm_6_Nce_6_ (Pep) and (b) Nbrpm_6_Nce_6_Nc_6_IR780 (PepIR).


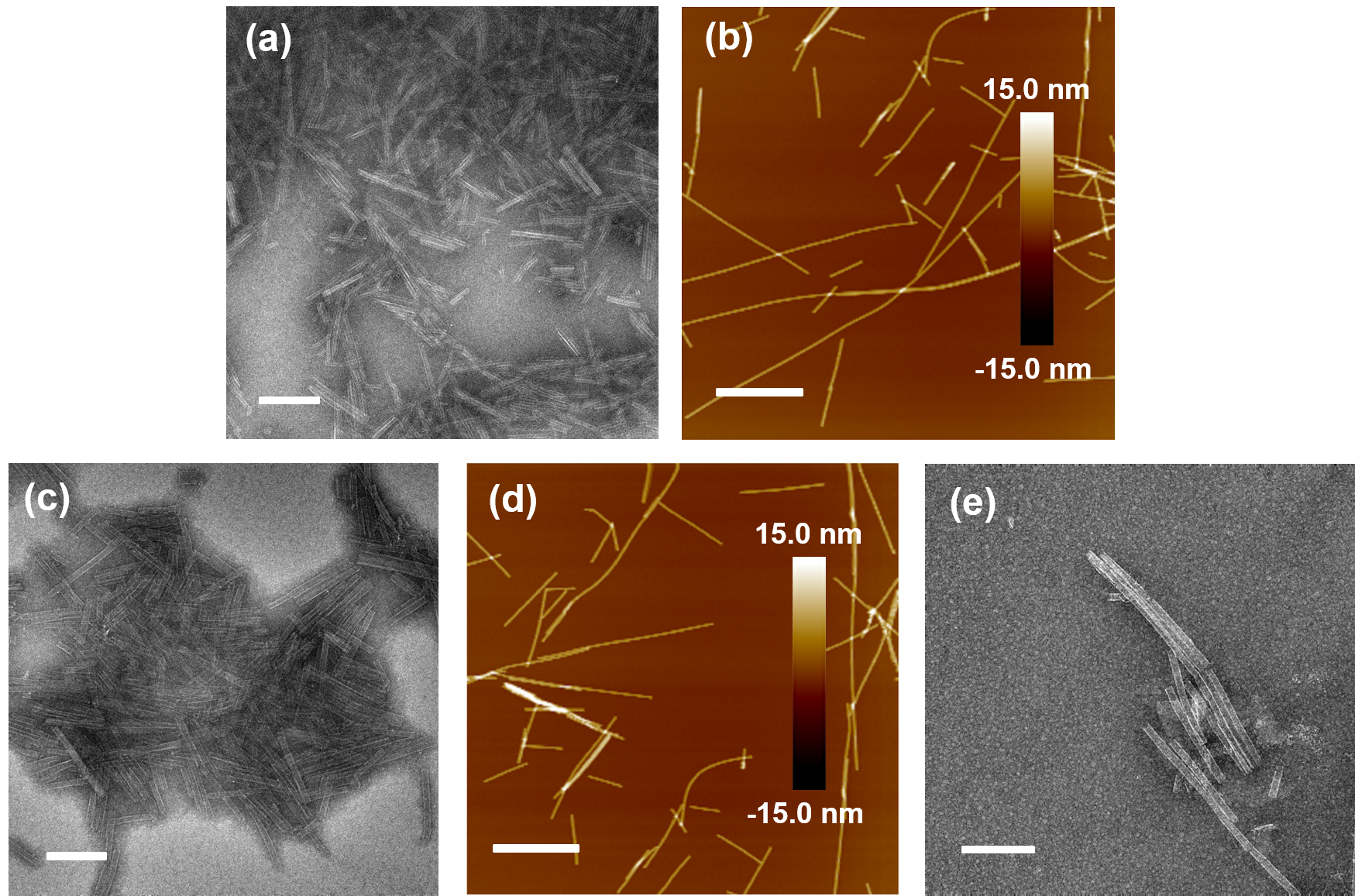


**Figure S2.** (a) TEM image of PepIR-20 nanotubes (scale bar, 500 nm). (b) *ex-situ* AFM image of PepIR-20 nanotubes (scale bar, 2.0 μm). (c) TEM image of PepIR-40 nanotubes (scale bar, 500 nm). (d) *ex-situ* AFM image of PepIR-40 nanotubes (scale bar, 2.0 μm). (e) TEM image of PepIR-100 nanotubes (scale bar, 200 nm).


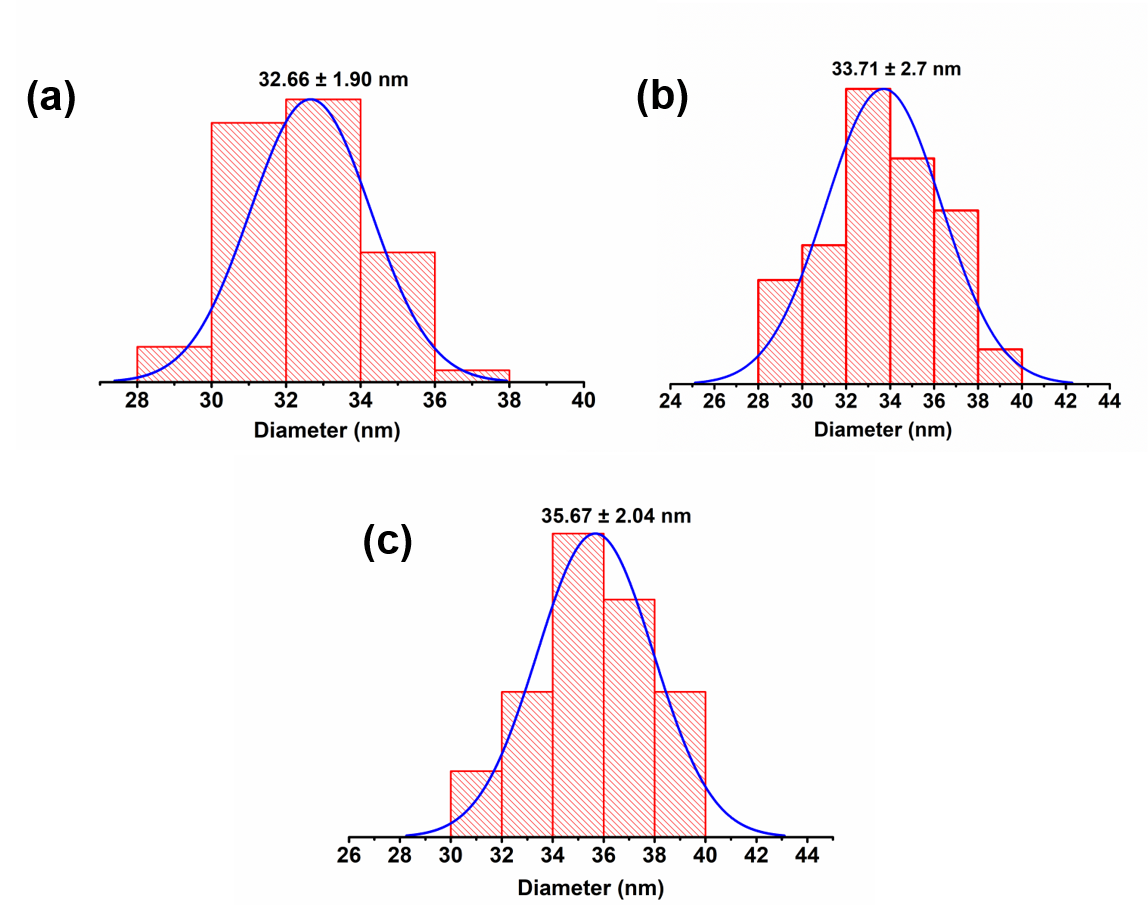


**Figure S3.** Statistical diameter distribution of PepIR-20 nanotubes (a), PepIR-40 nanotubes (b) and PepIR-100 nanotubes (c) measured from the TEM results. The number above histogram is the average tubular diameter; 50 nanotubes were analyzed for each size distribution.


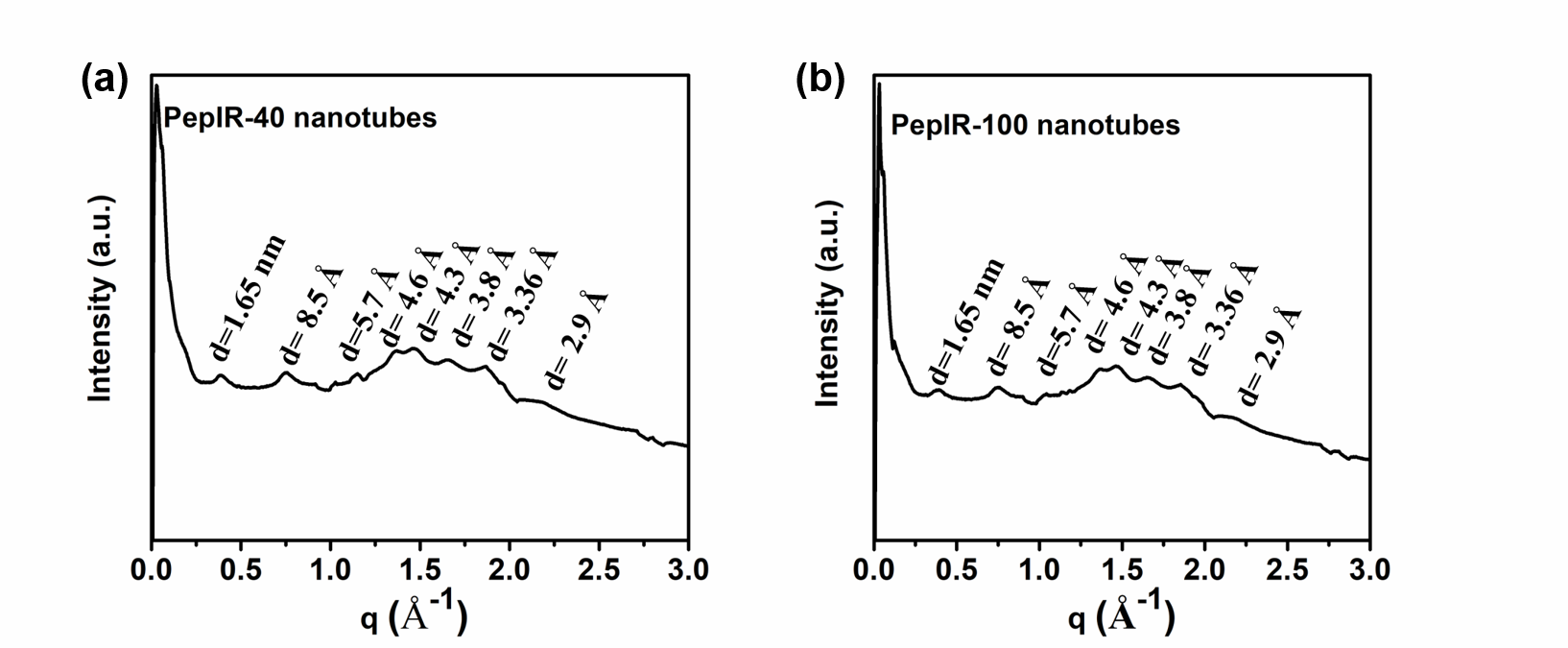


**Figure S4.** XRD data of PepIR-40 nanotubes (a) and PepIR-100 nanotubes (b). The values above each peak were calculated according to the formula of d=2π/*q*, and these numbers are similar to those of Pep nanotubes, indicating that PepIR-20, PepIR-40 and Pep-IR100 nanotubes all exhibited similar structures to those of Pep nanotubes.

**
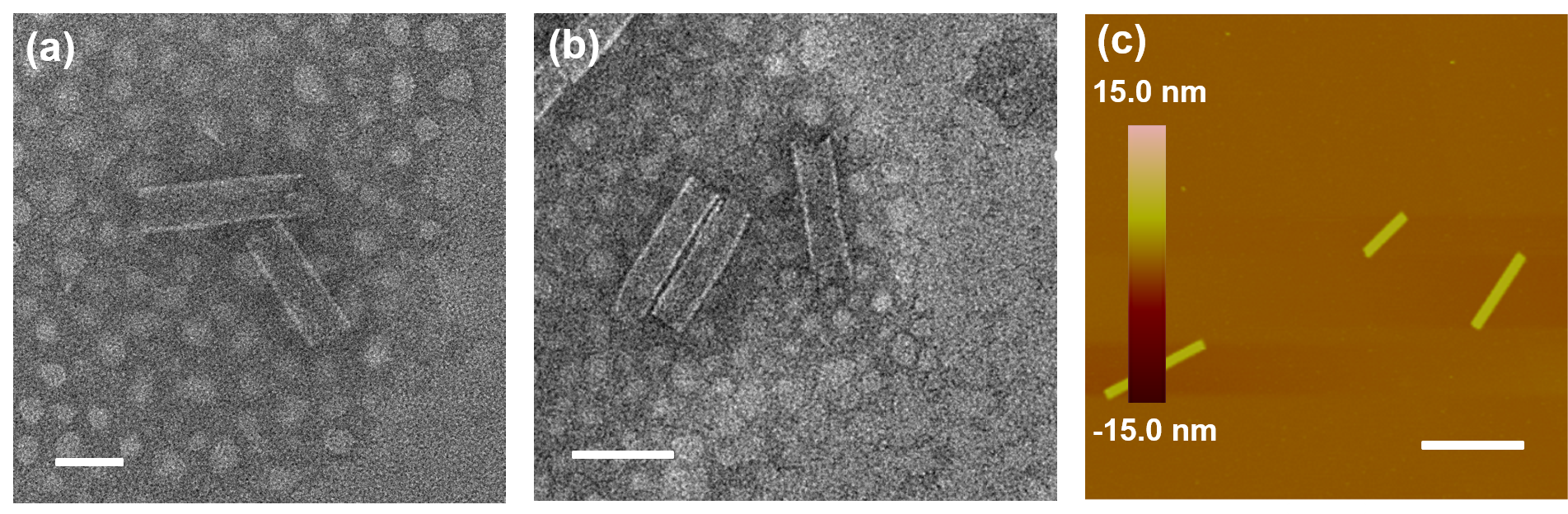
**

**Figure S5.** TEM images (a) (scale bar, 50 nm) (b) (scale bar, 100 nm) and *ex-situ* AFM image (scale bar, 500 nm) (c) of sc-PepIR-40 nanotubes as a result of ultrasonication.


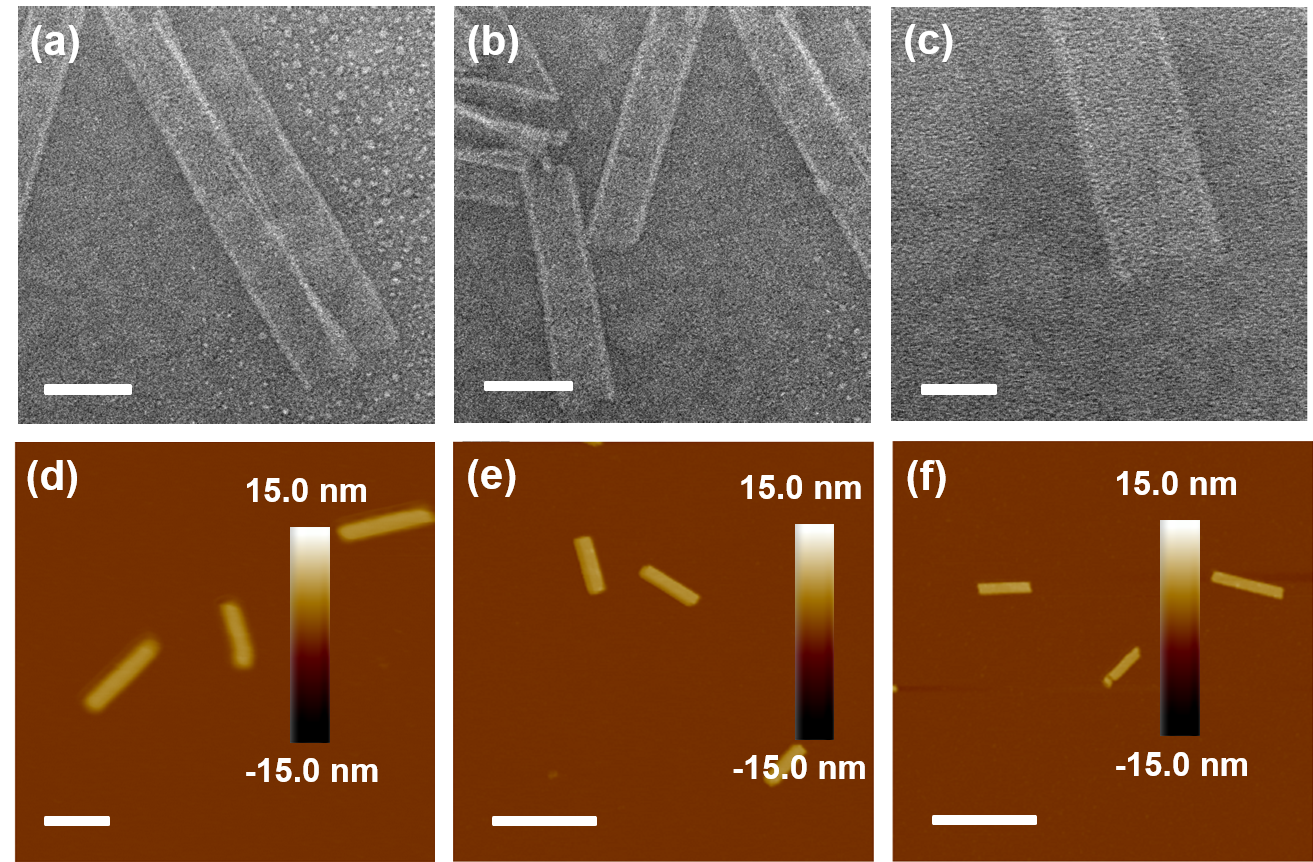
**Figure S6.** TEM images ((a, b), scale bar, 50 nm), ((c), scale bar, 20 nm) and *ex-situ* AFM images [(d), scale bar, 200 nm; (e, f), scale bar, 500 nm] of sc-PepIR-40 nanotubes after being treated at 50 °C. These results showed the high stability of these sc-PepIR-40 nanotube, which they remained the same tubular morphology after being incubated in aqueous solution at 50 °C for 6 h.


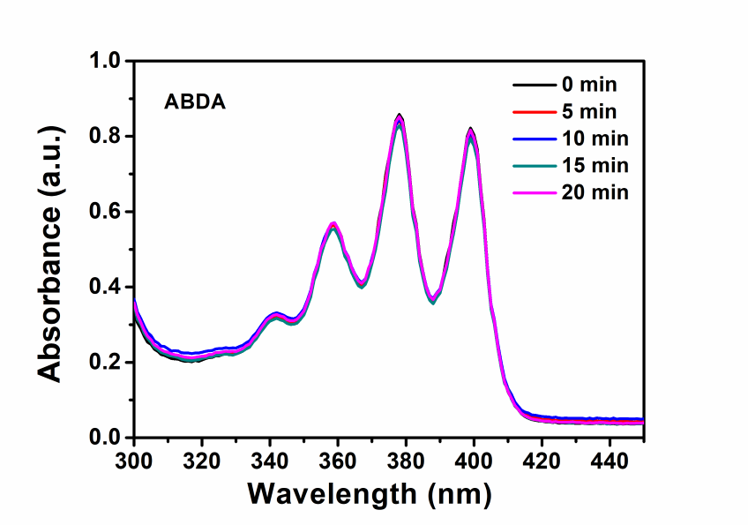


**Figure S7.** UV-vis absorption spectra of ABDA under 808 nm laser irradiation for different time. There was no obvious change in ABDA absorption after exposure to 808 nm laser irradiation.


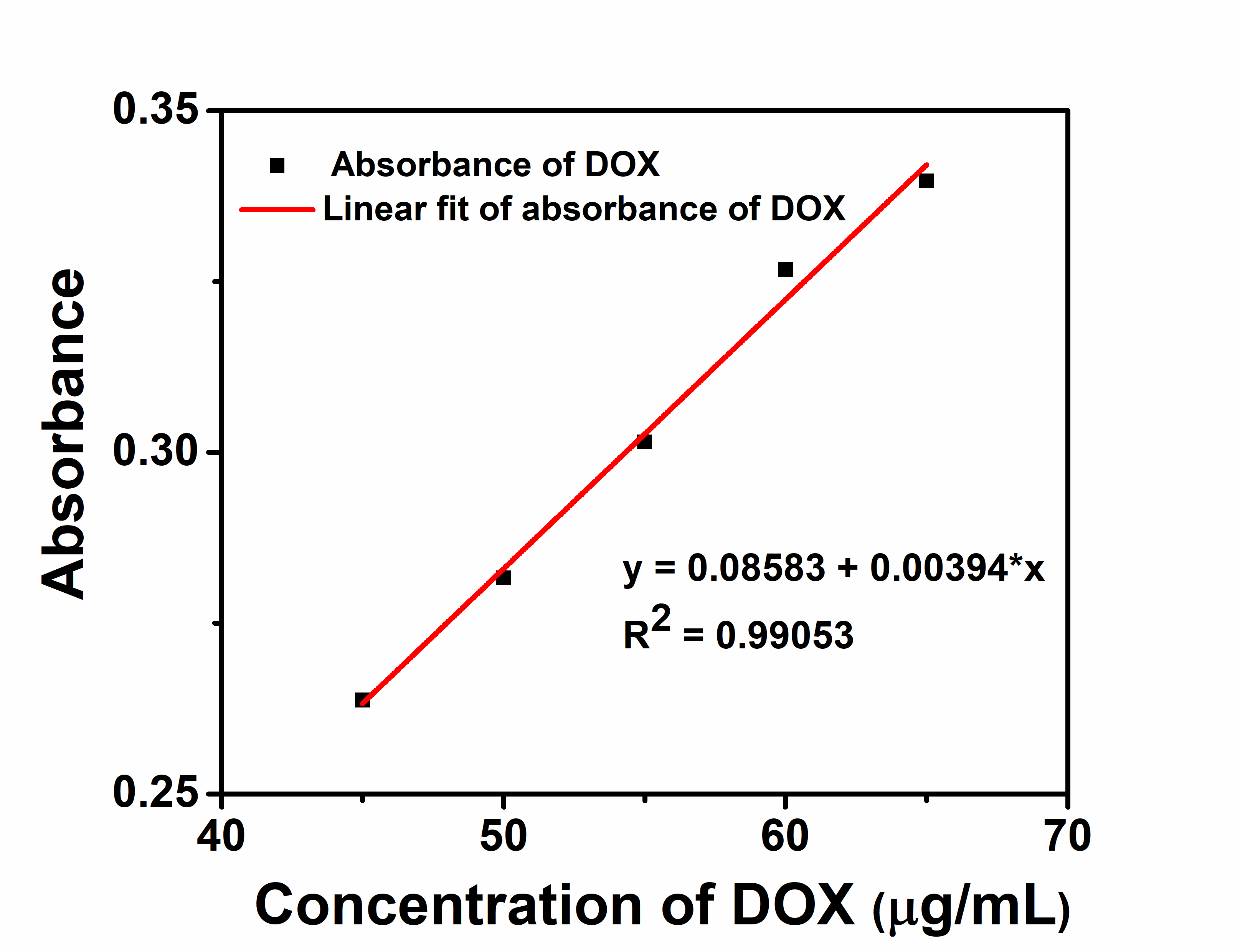


**Figure S8.** The calibration curve obtained from four DOX solutions at different concentrations.


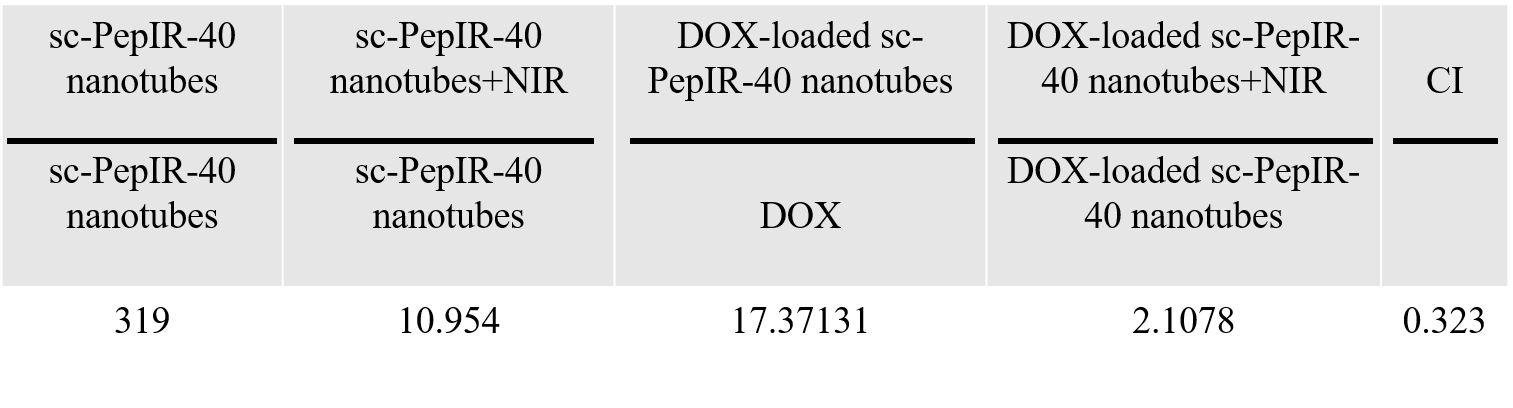


**Table S1.** The half-maximal inhibitory concentration IC_50_ (μM) of U87MG cells under different treatments. The first row shows the different treatments of U87MG cells; the second row shows the targets that we calculated IC_50_; the third row shows the values of corresponding IC_50_ (μM). The IC_50_ of DOX in chemotherapy and the IC_50_ of sc-PepIR-40 nanotubes in phototherapy are 17.37131 μM and 10.954 μM, respectively. A significant decrease of IC_50_ in combined therapy was observed (2.1078 μM). Moreover, the combination index (CI) was calculated to be 0.323 (<1), confirming a high synergistic therapeutic effect of chemotherapy and phototherapy.
